# TypA is involved in virulence, antimicrobial resistance and biofilm formation in *Pseudomonas aeruginosa*

**DOI:** 10.1186/1471-2180-13-77

**Published:** 2013-04-09

**Authors:** Anke Neidig, Amy TY Yeung, Thibaut Rosay, Beatrix Tettmann, Nikola Strempel, Martina Rueger, Olivier Lesouhaitier, Joerg Overhage

**Affiliations:** 1 Karlsruhe Institute of Technology (KIT), Institute of Functional Interfaces, PO Box 3640, Karlsruhe, 76021, Germany; 2 Centre for Microbial Diseases & Immunity Research, University of British Columbia, 2259 Lower Mall, Vancouver, BC, Canada; 3 Laboratory of Microbiology Signals and Microenvironment, LMSM EA 4312, University of Rouen, 55 rue Saint Germain, Evreux, 27000, France

**Keywords:** Pseudomonas aeruginosa, Pathogen, TypA, Type III secretion system, Virulence, Dictyostelium discoideum, Macrophage, Biofilm, Resistance

## Abstract

**Background:**

*Pseudomonas aeruginosa* is an important opportunistic human pathogen and is extremely difficult to treat due to its high intrinsic and adaptive antibiotic resistance, ability to form biofilms in chronic infections and broad arsenal of virulence factors, which are finely regulated. TypA is a GTPase that has recently been identified to modulate virulence in enteric Gram-negative pathogens.

**Results:**

Here, we demonstrate that mutation of *typA* in *P. aeruginosa* resulted in reduced virulence in phagocytic amoebae and human macrophage models of infection. In addition, the *typA* mutant was attenuated in rapid cell attachment to surfaces and biofilm formation, and exhibited reduced antibiotic resistance to ß-lactam, tetracycline and antimicrobial peptide antibiotics. Quantitative RT-PCR revealed the down-regulation, in a *typA* mutant, of important virulence-related genes such as those involved in regulation and assembly of the Type III secretion system, consistent with the observed phenotypes and role in virulence of *P. aeruginosa*.

**Conclusions:**

These data suggest that TypA is a newly identified modulator of pathogenesis in *P. aeruginosa* and is involved in multiple virulence-related characteristics.

## Background

*Pseudomonas aeruginosa* is a ubiquitous environmental Gram-negative soil bacterium that is also an important opportunistic human pathogen causing a variety of different nosocomial infections including pneumonia, catheter and urinary tract infections as well as sepsis in burn wound and immunocompromised patients [1]. Moreover, *P. aeruginosa* is the most prevalent and significant pulmonary pathogen in patients with cystic fibrosis causing eventually fatal lung disease [2]. The inability to successfully clear *P. aeruginosa* infections through antibiotic treatment is a major contributor to the complicated and often severe outcome of *P. aeruginosa* infections [3]. It demonstrates high intrinsic resistance to antibiotics and an ability to develop even higher resistance through mutation, acquisition of genetic elements, and adaptation to environmental conditions, e.g. through biofilm formation on surfaces.

*P. aeruginosa* also possesses a large arsenal of virulence-related factors. Among others are a type II, III and VI secretion system and their associated effector proteins such as extracellular proteases and phospholipases and the Type III secreted toxins ExoU, S, T and Y. In addition, they have flagella and type IV pili that are involved in motility and host cell adhesion [4-6]. *P. aeruginosa* also regulates the gene expression of most virulence factors including genes involved in iron acquisition (e.g. pyoverdine), toxin production (hydrogen cyanide), exopolysaccharide biosynthesis or biofilm formation in a cell density dependant manner termed quorum sensing mediated by the two master regulators LasR and RhlR [4,7,8]. Although some virulence factors seem to be host or site specific, the majority are involved in multi-host infections in a variety of different non-mammalian and mammalian organisms including amoebae, flies, nematodes, rodents and humans [9-11].

The coordinated control of the production of virulence and antibiotic resistance factors and the ability to adapt to various environmental changes is a likely and important reason that *P. aeruginosa* is a successful and common pathogen. The genome sequence of this microorganism revealed that more than 500 genes, representing nearly 10% of the genome, have a putative role in regulation [1]. In addition to conventional regulators involved in transcription of particular genes, e.g. sigma factors, repressors, activators or two-component response regulators, *P. aeruginosa* possesses several additional proteins that modulate translation, protein biosynthesis and degradation, etc. Here we have defined the role of the GTPase TypA in the lifestyle of *P. aeruginosa*.

TypA, also named BipA, belongs to a superfamily of ribosome-binding GTPases within the TRAFAC class (translation factors) of GTPases [12-14]. GTPases are widely distributed molecular switches found across all bacterial species, and generally cycle between a GDP-bound “off” state and a GTP-bound “on” state [14,15]. Collectively they are involved in the regulation of multiple cellular processes and can play important roles in translation, ribosome biogenesis and assembly, tRNA modification, protein translocation, cell polarity, cell division and signaling events [14,16]. Since GTPases are widely conserved in prokaryotes and play an essential role in many important bacterial processes, they are an attractive target for novel antibiotic development [17].

TypA is highly conserved in bacteria and shares sequence homologies to other GTPases like elongation factor G. It is found in many pathogens of significant public health importance including *Vibrio cholera*, *Yersinia pestis* and *Mycobacterium tuberculosis*[13]. Although its precise function is still poorly understood, TypA has been suggested to be involved in the regulation of virulence and stress responses in pathogenic *Escherichia coli*[18,19] and *Salmonella enterica* Serovar Typhimurium [15], and stress responses in non-pathogenic *Sinorhizobium meliloti*[20] and *Bacillus subtilis*[21]. Open reading frame PA5117 is annotated as the GTPase TypA, exhibits 75% sequence homology to TypA/BipA from *E. coli*[13], and plays a role in swarming motility and biofilm formation in *P. aeruginosa* PAO1 [22]. However, the role of TypA in pathogenesis of *P. aeruginosa* is still unknown.

Here we constructed a knock-out mutant of *typA* in *P. aeruginosa* PA14 and demonstrated the involvement of TypA in the pathogenesis of *P. aeruginosa* using different *in vitro* and *in vivo* infection model systems. Consistent with these data, we showed using gene expression analysis that several virulence-associated genes were down-regulated in a TypA mutant during host-pathogen interaction. We also found that TypA plays a role in antibiotic resistance to a variety of different antibiotics and initial attachment leading to subsequent biofilm formation in *P. aeruginosa* PA14.

## Results

### TypA is involved in *P. aeruginosa* virulence

Previously, we showed that a mutation in the *typA* gene led to a defect in particular virulence-associated features such as swarming and biofilm formation in *P. aeruginosa* PAO1 [22]. To further investigate the involvement of TypA in the pathogenesis of *P. aeruginosa*, we constructed a site-directed *typA* knock-out mutant in *P. aeruginosa* strain PA14. Strain PA14 is capable of infecting a wide range of organisms including the amoeba *D. discoideum*[23,24] and the nematode *C. elegans*[4] and was therefore more suitable for virulence analysis using *in vivo* model systems in comparison to strain PAO1.

**Figure 1:**
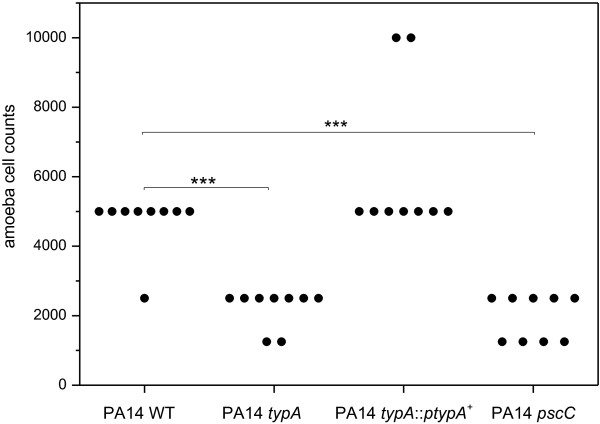
***D. discoideum *plate killing assay.** Each point represents the number of amoebae required to form a plaque on the bacterial lawn of *P. aeruginosa* PA14 strains after 5 days of incubation. The *typA* and *pscC* mutants had a major defect in this virulence model of infection, which was statistically significant as measured with the Mann Whitney test (*** *p* < 0.001, n = 9).

Detailed analyses of virulence attenuation of the PA14 *typA* mutant using the unicellular eukaryotic model organism *D. discoideum* revealed a consistent, statistically significant (*P* < 0.001 by Mann Whitney test) 2-fold reduction in the numbers of amoebae required to form a plaque when compared to wild type strain PA14 (Figure 1). The virulence phenotype could be completely restored to wild type level by heterologous expression of the cloned *typA* gene in strain PA14 *typA*::p*typA*^*+*^. In comparison, a similar 2-fold reduction in numbers of amoebae was determined when analyzing PA14 transposon mutant ID29579 obtained from the Harvard PA14 mutant library [25] with a defect in the *pscC* gene, which is an essential part of the Type III secretion system machinery [26], as a control (Figure 1). To exclude the fact that a simple growth deficiency of the *typA* mutant is responsible for the attenuated virulence phenotype of PA14 *typA*, we performed growth analyses at 23°C and 37°C in M9 minimal medium using a Tecan plate reader under shaking conditions. At both temperatures no significant growth defect was observed (data not shown).

**Figure 2:**
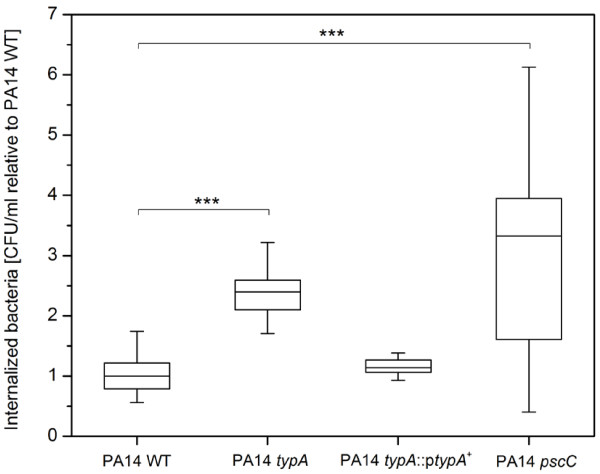
**Uptake of *P. aeruginosa *by human macrophages.** Strains were incubated with 1.5 × 10^5^ cells/ml macrophages for 1 h at an MOI of 10. Subsequently, extracellular and attached bacteria were killed by treatment with gentamicin, and macrophages were lysed with Triton X-100 and lysates plated to enumerate viable intracellular bacteria. Results are expressed as the percentage of intracellular bacteria that were recovered relative to the PA14 WT. The box plots (median, thick line in the box) represent the mean of 3 independent biological repeats, each assayed minimum in duplicate (n = ≥6). *** indicates a statistically significant difference (*p* < 0.001), between the *typA* and *pscC* mutant and PA14 WT as determined by Whitney Mann test.

Since phagocytosis of pathogens by macrophages is a crucial factor in the human immune defense system, we quantitatively analyzed *in vitro* uptake of PA14 WT and respective mutant strains using human macrophages in a gentamicin protection assay. We determined a more than 2-fold increase in internalization of the *typA* and the *pscC* mutant strain in comparison to cells of PA14 WT and complemented strain PA14 *typA*::p*typA*^*+*^ (Figure 2). This result was in accord with the virulence defect observed in the amoeba model of infection, which is similarly based on phagocytic killing of bacterial cells.

**Figure 3:**
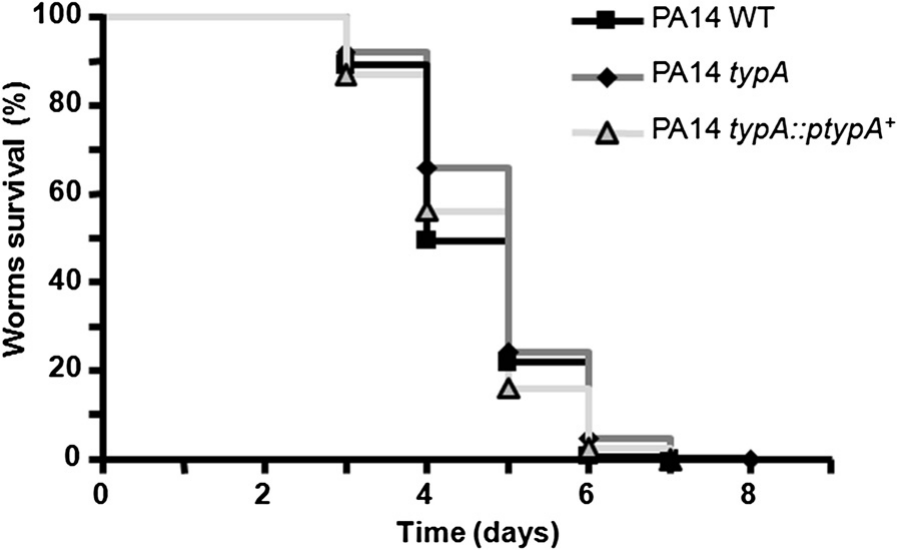
***P. aeruginosa *virulence towards *C. elegans *worms.** (**a**) Slow killing: Kaplan-Meier survival plots of worms fed with *P. aeruginosa* PA14 control (n = 320) (squares), PA14 *typA* mutant (n = 277) (diamonds) and the complemented strain PA14 *typA*::p*typA*^+^ mutant (n = 319) (triangles). Each value reported for the assay is the mean of measurements of nine samples from three independent experiments.

To better understand the mechanism of virulence deficiency in the *typA* mutant, we additionally determined virulence in a nematode infection model using *C. elegans* as host organism under slow killing conditions. In contrast to the Type III secretion based killing of unicellular eukaryotic hosts like amoebae or macrophages, nematode killing is rather dependent on quorum sensing related virulence features in *P. aeruginosa*[4,27]. When feeding *C. elegans* with PA14 wild type, *typA* mutant and complemented PA14 *typA*::p*typA*^*+*^ strain, we observed a similar worm killing rate for all tested strains with only marginal differences between PA14 wild type and *typA* knock-out mutant at day 4 of the incubation time (Figure 3).

### TypA is involved in rapid attachment and biofilm formation

**Figure 4:**
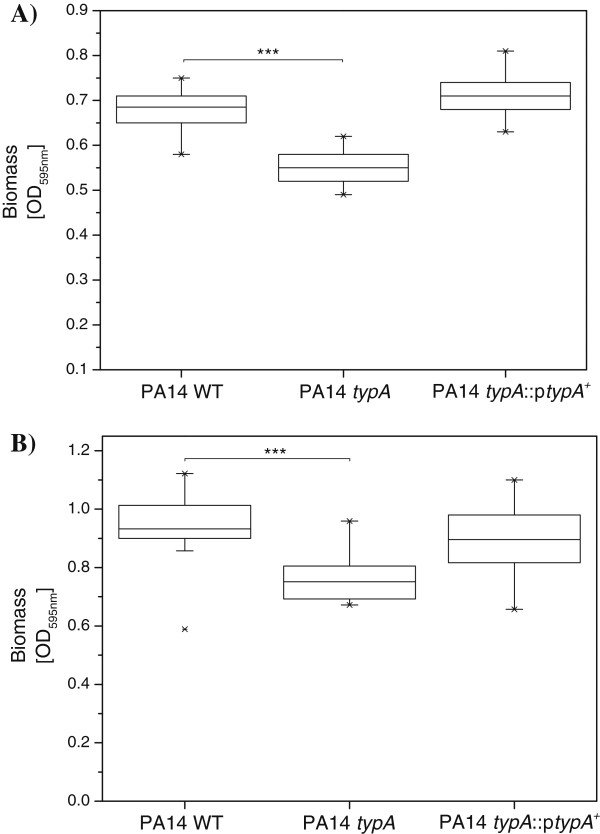
**Defects in attachment and biofilm formation in the *typA *mutant.** (**A**) Requirement for *typA* in rapid attachment. Attachment was determined using diluted overnight cultures for 60 min at 37°C. Adhered cells were stained with crystal violet. (**B**) Requirement for *typA* in static biofilm formation. Cells were grown for 24 h at 37°C in polystyrene microtiter plates containing BM2 medium with 0.5% (w/v) casamino acids. Microtiter wells were washed several times during incubation to remove planktonic bacteria. Adherent biofilms were stained with crystal violet, followed by ethanol solubilization of the crystal violet and quantification (*A*_595nm_) of stained wells. The box plots (median, thick line in the box) represent the mean of 3 independent biological repeats, each assayed in quintuplicate (n = 15). *** indicates a statistically significant difference (*p* < 0.001), between the *typA* mutant and PA14 WT as determined by Whitney Mann test.

The ability to form biofilms is a known and important factor in the pathogenesis of *P. aeruginosa*. To assess the ability of the *typA* mutant to develop biofilms, static microtiter assays were performed to show that PA14 *typA* displayed with approximately 20% reduction a statistically significant (*P* < 0.001 by Mann Whitney test) impairment in biofilm formation at 24 hours (Figure 4) in comparison to the PA14 WT. This biofilm defect could be complemented by heterologous expression of wild type *typA* in strain PA14 *typA*::p*typA*^+^. To analyze whether this biofilm formation phenotype emerged during the initial adherence phase or later during biofilm growth, a rapid attachment assay was carri d out. The mutant PA14 *typA* exhibited with approximately 20% reduction a statistically significant (*P* < 0.001 by Mann Whitney test) defect in adherence which was similar to the biofilm phenotype.

However, the investigation of flagellum-mediated swimming and swarming motility as well as the type IV pilus-mediated twitching motility, which are all involved in attachment and subsequent biofilm development, revealed no differences between mutant and wild type strain (data not shown) ruling out defects in the biosynthesis and function of these cellular appendages in the *typA* mutant.

### Antibiotic susceptibility testing

Since recent studies have demonstrated a role for TypA in multidrug resistance in *E. coli*[28], we studied the impact of the *typA* gene in antibiotic resistance of *P. aeruginosa* against a variety of different antimicrobial compounds. As shown in Table 1, the *typA* mutant exhibited a consistent 2-fold increase in susceptibility to the cationic peptides polymyxin B and colistin, the ß-lactam antibiotics ceftazidime and meropenem, as well as tetracycline in comparison to the parent strain. This altered susceptibility could be complemented by introducing wild type copies of *typA* into the mutant strain. No change in susceptibility was observed regarding the fluoroquinolone ciprofloxacin, the aminoglycoside tobramycin, and the cationic host defence peptide LL-37 (Table 1).

**Table 1:** **MICs of different antibiotics towards *P. aeruginosa *PA14 WT, PA14 *typA *mutant and complemented mutant PA14 *typA*::p*typA***^**+a**^

	**MIC (μg/ml)**		
**Antibiotic**	**PA14 WT**	**PA14*typA***	**PA14*typA*::p*typA***^**+**^
Ciprofloxacin	0.03	0.03	0.03
Meropenem	2	1	2
Ceftazidime	4	2	4
Tetracycline	8	4	8
Tobramycin	0.25	0.25	0.25
Polymyxin B	0.5	0.25	0.5
Colistin	0.25	0.125	0.25
LL-37	16	16	16

^a^MICs were determined by serial 2-fold dilutions in MH-medium. The MIC represents the concentration at which no growth was visually observed after 24 h of incubation at 37°C. The values shown are the modes of 4 to 6 independent experiments.

### Reduced virulence of PA14 *typA* due to down-regulation of the Type III secretion system

Previous studies have shown, that uptake by and killing of eukaryotic host cells is highly dependent on the Type III secretion system in *P. aeruginosa*[5,29,30]. To analyze the potential molecular basis for reduced virulence of the *typA* mutant observed in our experiments, we investigated gene expression of known virulence-associated genes in *P. aeruginosa* using qRT-PCR on bacterial RNA of wild type and *typA* mutant strain isolated during host-pathogen interaction with *D. discoideum*. These studies revealed that under the tested conditions, genes coding for the synthesis, function and regulation of the Type III secretion system, were significantly down-regulated in the *typA* mutant compared to wild type (Table 2). This observed down-regulation of important virulence-related genes is consistent with the noticed virulence defects in the cellular infection studies with *D. discoideum* and human macrophages as hosts.

**Table 2:** **Gene expression of selected Type III secretion genes in the*typA*mutant compared to that in wild type PA14 using RT-qPCR**

**Gene**	**Fold change in gene expression in the *typA *mutant relative to wild type**^**a**^
**T3SS**	
*exsA*	−3.1 ± 0.5
*pscC*	−2.3 ± 0.4
*pscJ*	−3.5 ± 0.3
*pscT*	−5.1 ± 0.3
*pcrV*	−5.8 ± 0.6

## Discussion

In this study, we have shown that TypA is involved in virulence of *P. aeruginosa* by analyzing the consequences of a *typA* knock-out on phagocytic amoebae and human macrophages as well as the interaction with the nematode *C. elegans*. Moreover, TypA also contributes to resistance to different antibiotics as well as attachment and biofilm formation in *P. aeruginosa*.

TypA is a highly conserved prokaryotic GTPase exhibiting structural homologies to translation factor GTPases such as EF-G and LepA and is described to associate with the ribosomes under normal bacterial growth [15,31]. In enteropathogenic *E. coli* (EPEC), TypA co-ordinates the expression of key stress and virulence factors including flagella, Type III secretion system as well as the LEE and the espC pathogenicity islands [18,32] by regulating gene expression of major regulators such as Ler, which in turn controls these respective pathogenicity islands. Consequently, it has been suggested that TypA is on a relatively high level in the complex regulatory hierarchy of virulence regulation in this organism [18,32]. In contrast, analysis in *Mycobacterium tuberculosis* revealed that TypA does not act as a virulence regulator in this human pathogen, ruling out a general involvement of this protein in virulence regulation in pathogenic bacteria [33]. However, our results demonstrate that TypA plays a role in the pathogenesis of *P. aeruginosa.* The *typA* knock-out mutant exhibited a significant virulence deficiency in both the amoebae infection model and the macrophage uptake studies. These defects were comparable to a *pscC* mutant with a disrupted Type III secretion system and consistent with the down-regulation of Type III secretion genes during host-pathogen interaction. The Type III secretion system of Gram-negative bacteria is an important factor of pathogenesis and is involved in manipulating eukaryotic cells by injecting effector proteins into the host [27] and impacts diretly on bacterial uptake by phagocytic cells [30]. In *P. aeruginosa*, this complex, needle-like machinery is encoded by 36 genes and an important factor for the survival during interaction with phagocytic amoebae or human macrophages, among others [5,29,30]. Using qRT-PCR, we observed the down-regulation of selected genes participating in different aspects of Type III secretion, including *pscC* (outer membrane ring), *pscJ* (basal substructure), *pscT* (translocation), and *pcrV* (needle-tip complex). The transcription of Type III secretion genes is tightly regulated by ExsA in *P. aeruginosa*. This master regulator controls both, the synthesis of the secretion system as well as effector protein production, and interacts in concert with the global cyclic AMP and Gac regulatory systems [5,34]. Our studies showed that in addition to genes involved in assembly of the secretion apparatus, expression of *exsA* was also significantly down-regulated in the *typA* mutant compared to wild type cells. To identify, if increasing Type III secretion activity is sufficient to complement our virulence phenotype, we heterologously expressed the *exsA* gene using plasmid pUCP20::*exsA*^*+*^ in the *typA* mutant and obtained an identical number of amoebae required for plaque formation in both mutant and wild type PA14 harboring pUCP20::*exsA* (data not shown). These findings suggest that, like in *E. coli*, TypA is part of the complex regulatory cascade involved in controlling Type III secretion in *P. aeruginosa* by impacting expression of genes involved in regulation and assembly of the secretion machinery. Since TypA is a GTPase associated with the ribosomes, a further down-regulation of the Type III secretion machinery at the translational level might also be possible; this could result in an even stronger impairment of the Type III secretion system.

Previously, it has been shown that the Type III secretion system including its associated virulence effectors does not play a noticeable role in nematode killing [4,35], which is rather dependent on quorum sensing related virulence factors such as RhlR and LasR [27,36]. Thus, it is not surprising, that a mutation in *typA* with a down-regulation in the Type III secretion system did not result in significant virulence attenuation in our studied infection model. Additional analyses of quorum sensing dependent production of the extracellular protease LasB and toxin pyocyanin did not reveal a significant difference between wild type and mutant strain (data not shown) demonstrating that TypA does, most likely, not affect quorum sensing in *P. aeruginosa* PA14.

TypA was first described to be involved in human bactericidal/permeability-increasing protein BPI, a cationic host defence peptide from human neutrophils, resistance in *S. typhimurium* and *E. coli*[37,38]. Although we were not able to detect any differences regarding resistance to cationic human host defence peptide LL-37, we found that TypA is also participating in resistance against a variety of clinically important antibiotics such as ß-lactam, tetracycline and peptides antibiotics in *P. aeruginosa*. Due to this wide range of different antimicrobials with unrelated modes of action, it is likely that the involvement of TypA in antibiotic stress resistance is rather unspecific and could be based on the fact that TypA is part of a more general stress response resulting in resistance. This would be in accordance with earlier studies showing the involvement of TypA in a wide variety of very different stress responses in a number of pathogenic and non-pathogenic microorganisms, among other stress factors were antimicrobials, low pH, oxidative or detergent stress [20,37,38].

Biofilm formation is a crucial factor in the pathogenesis of *P. aeruginosa* and is involved in many chronic infections including chronic lung infections of cystic fibrosis patients or foreign body part infections [39]. Biofilm development is a sequential process initiated by the attachment of planktonic cells to a surface, followed by formation of microcolonies and biofilm maturation. Bacteria grown in biofilms exhibit high resistance against antimicrobial agents, are protected from the host immune response and are notoriously difficult to eradicate [39-41]. Although the *typA* mutant was able to form biofilms, we observed a more than 20% reduction in biofilm mass compared to wild type cells. By analyzing the initial adhesion phase of biofilm development, we identified that this reduction in biofilm is, at least in parts, due to a significant impairment in rapid attachment of the *typA* mutant in the respective microtiter plate assay. This impairment in attachment results in less bacterial cells initiating biofilm formation and subsequently lower biofilm growth, which could not be restored to wild type levels during further biofilm development. Interestingly, it was shown previously that TypA is involved in adherence to biotic surfaces and interaction of enteropathogenic *E. coli* with epithelial cells [19] and the symbiotic interaction of *S. meliloti* with the nodules of the legume *Medicago truncatula*[20] indicating a role of TypA in cell-cell contact. Biofilm initiation and cell adhesion are rather complex processes influenced by a large number of proteins and factors, among others are flagellum- and type IV pilus-mediated bacterial motility and attachment, respectively. Although we have recently shown, that TypA is involved in swarming motility in *P. aeruginosa* strain PAO1 [22], we did not observe any impairment in swimming, swarming or twitching motility in the PA14 *typA* mutant suggesting a mechanism not related to a defect in flagella or type IV pili biogenesis and function, respectively, is responsible for the impairment in adhesion and biofilm initiation in this mutant.

## Conclusions

In this study, we were able to demonstrate the involvement of TypA in the pathogenesis of *P. aeruginosa* by analyzing the consequences of a *typA* knock-out*.* This *typA* mutant exhibited reduced virulence towards phagocytic amoebae and increased uptake by human macrophages, impaired cell attachment and subsequent biofilm formation and a reduction in antimicrobial resistance to ß-lactam, tetracycline and antimicrobial peptide antibiotics. The *typA* mutant exhibited a dysregulation of genes involved in regulation and assembly of the Type III secretion system, consistent with the observed phenotypes and role in virulence regulating Type III secretion system.

## Methods

### Organisms, plasmids, primers, and growth conditions

The organisms and plasmids used in this study are listed in Table 3 and include *P. aeruginosa* PA14 [25] and *Dictyostelium discoideum* Ax2 [24]. The sequences of DNA primers (Eurofins MWG Operon, Germany) used in these studies are available upon request. *E. coli* was routinely grown in Luria-Bertani (LB) broth, *P. aeruginosa* in M9 [23], LB or BM2 [44] medium, and *D. discoideum* in HL5 broth medium [45]. *D. discoideum* was incubated in cell culture flasks (Greiner Bio One, Frickenhausen, Germany) at 22.5°C and sub-cultured twice a week. When required for plasmid or resistance gene selection or maintenance, gentamicin, ampicillin and carbenicillin were added at final concentrations of 30, 100 and 200 μg/ml, respectively.

**Table 3:** Strains and plasmids used in this study

**Strain or plasmid**	**Description and characteristics**^**a**^	**Reference**
**Strains**		
***P. aeruginosa***		
PA14 WT	Wild type *P. aeruginosa* PA14	[25]
PA14 *typA*	*typA* insertion mutant of PA14, Gm^r^	This study
PA14 *typA*::p*typA*^*+*^	Complemented mutant PA14 *typA* harboring plasmid pUCP20::*typA*^*+*^; Gm^r^, Cb^r^	This study
PA14 *pscC*	*pscC* transposon mutant ID29579 of the Harvard PA14 mutant library	[25]
***E. coli***		
DH5α	F^–^ϕ80*lacZ*ΔM15 Δ(*lacZYA-argF)U169 deoR recA1 endA1 hsdR17*(r_K_^–^ m_K_^+^) *supE44*λ^–^*thi-1 gyrA96 relA*	Invitrogen
**Plasmids**		
pUCP20	*E. coli – Pseudomonas* shuttle vector for constitutive expression of cloned genes, Cb^r^	[42]
pEX18Ap	Suicide vector for mutant regeneration in *Pseudomonas*, Amp^r^/Cb^r^	[43]
pUCP20::*typA*^*+*^	pUCP20 containing the cloned *typA* gene; Amp^r^/Cb^r^	This study
pUCP20::*exsA*^*+*^	pUCP20 containing the cloned *exsA* gene; Amp^r^/Cb^r^	This study

^a^ Antibiotic resistance phenotypes: Amp^r^, ampicillin resistance for *E. coli*; Cb^r^, carbenicillin resistance for *P. aeruginosa*; Gm^r^, gentamicin resistance.

### Amoeba plaque assay

In this cellular model system, a more virulent *P. aeruginosa* strain will limit the ability of the amoebae to form a plaque on a bacterial lawn to a greater extent than a less virulent strain. The assay was performed according to the method described previously [23]. Briefly, 50 μl of overnight cultures grown in LB medium were mixed with 200 μl PBS buffer and plated on M9 agar plates. Plates were dried on a laminar flow bench for 15 min to obtain a dry, even bacterial lawn. Amoebae grown for 2 to 4 days in the respective medium were harvested by centrifugation at 510 x g for 10 minutes, washed and resuspended in PBS buffer. Cells were adjusted to 8 × 10^6^ cells per ml and kept on ice. This stock solution was serially diluted and used to prepare droplets of 5 μl containing between 5 and 20,000 amoebae, which were subsequently spotted onto the bacterial lawn. Plates were incubated for 5 days at 22.5°C and the highest dilution at which growth of the amoebae caused a visible plaque of bacterial clearance was reported. Three independent experiments performed at least in duplicate were carried out for each bacterial strain.

### Gentamicin protection assay

*In vitro* internalization of PA14 WT and mutant cells by human macrophages derived from monocytes (MDM) was performed as previously described [46] with modifications. Briefly, mid-logarithmic phase cultures of *P. aeruginosa* were washed with complete RPMI medium and resuspended in 1 ml of the medium. The resuspended bacteria were added to 1.5 x 10^5^ MDM cells/ml, at a multiplicity of infection (MOI) of 10, and incubated for 1 h at 37°C. Subsequently, cells were washed with complete RPMI and incubated with 400 μg/ml of gentamicin for 30 min at 37°C to kill the extracellular and attached bacteria. After gentamicin treatment, MDM cells were washed and lysed with 0.1% Triton X-100. Lysates were plated onto LB agar and incubated overnight at 37°C. The next day, colonies were counted and relative phagocytic uptake was determined by CFU counts. Three independent experiments with at least duplicates in each experiment were performed for each bacterial strain.

### *Caenorhabditis elegans* synchronization and virulence assay

The *C. elegans* wild-type Bristol strain N2 was obtained from the *Caenorhabditis* Genetics Center (Minneapolis, MN, USA). *C. elegans* were maintained under standard culturing conditions at 22°C on nematode growth medium (NGM: 3 g NaCl, 2.5 g peptone, 17 g agar, 5 mg cholesterol, 1 ml 1 M CaCl_2_, 1 ml 1 M MgSO_4_, 25 ml 1 M KH_2_PO_4_, H_2_O to 1 liter) agar plates with *E. coli* OP50 as a food source [47]. Synchronous cultures of worms were generated after worm adult population exposure to a sodium hypochlorite/sodium hydroxide solution as previously described [48] and adapted [49]. The resulting eggs were incubated at 22°C on an *E. coli* OP50 lawn until the worms reached the L4 (48 hours) life stage (confirmed by light microscopy). Bacterial lawns used for *C. elegans* survival assays were prepared by spreading 50 μl of *P. aeruginosa* strains on 35 mm NGM conditioned Petri dishes supplemented with 0.05 mg ml−^1^ 5-fluoro-2^′^-deoxyuridine. This nucleotide analog blocks the development of the next *C. elegans* generation by inhibition of DNA synthesis. The plates were incubated overnight at 37°C and then placed at room temperature for 4 h. Fifteen to twenty L4 synchronized worms were harvested by resuspension in M9 buffer (3 g KH_2_PO_4_, 6 g NaHPO_4_, 5 g NaCl, 1 ml 1 M MgSO_4_, H_2_O to 1 liter), plated on the 35 mm assay Petri dishes and incubated at 22°C. Worm survival was scored after 1 h, 24 h and on each subsequent day, using an Axiovert S100 optical microscope (Zeiss, Oberkochen, Germany) equipped with a Nikon digital Camera DXM 1200 F (Nikon Instruments, Melville, NY, USA). Worms were considered dead when they remained static without grinder movements for 20 s. The results were expressed as the percentage of living worms and were the average of three independent assays performed in triplicate.

### Growth curves

Overnight cultures grown in LB medium were diluted into M9 medium to obtain equal starting optical densities at 600 nm (OD_600_). Five-μl portions of these cultures were added to 195 μl of fresh M9 medium in 96-well microtiter plates. The growth of the cultures at 37°C and 23°C under shaking conditions was monitored with a Tecan Infinite F200 Pro.

### Plasmid and *typA* knock-out mutant generation

For the construction and complementation of a *typA* knock-out mutant in *P. aeruginosa* PA14 the *typA* gene (gene number PA_67560) was amplified by PCR using *Eco*RI and *Hin*dIII flanked oligonucleotides, respectively, and subsequently cloned behind the *lac* promoter in the broad host range vector pUCP20, resulting in pUCP20::*typA*^+^. For the heterologous expression of the *exsA* gene, *exsA* was amplified by PCR using *Eco*RI and *Xba*I flanked oligonucleotides, respectively, and subsequently cloned into pUCP20, resulting in pUCP20::*exsA*^+^. These plasmids were then transferred into *E. coli* DH5α by transformation or *P. aeruginosa* by electroporation. The knock-out mutant was obtained according to the methods described previously [43]. Briefly, the hybrid plasmid pUCP20::*typA*^+^ was digested with *Sma*I to delete a 1.1 kb fragment from the *typA* gene, which was subsequently replaced with a Ω gentamicin resistance gene cassette for selection. The disrupted *typA*ΩGm gene was amplified by PCR and cloned into the suicide vector pEX18Ap [43] and transferred into *P. aeruginosa* PA14 to generate the *typA* knock-out mutant named *P. aeruginosa* PA14 *typA* by allelic exchange.

### MIC determination

MICs were measured using standard broth microdilution procedures [50] in Mueller Hinton (MH) medium. Growth was scored following 24 h of incubation at 37°C.

### Motility, biofilm and rapid attachment assays

Swimming, swarming and twitching motility were evaluated as described previously [44]. The abiotic solid surface assay was used to measure biofilm formation according to the previously described method with the following modifications [51]. Overnight cultures were diluted 1:100 in BM2 containing 0.5% (w/v) casamino acids and inoculated into 96-well polystyrene microtiter plates and incubated at 37°C for 60 min without shaking to allow bacterial cell adhesion. Subsequently, the microtiter wells were washed twice to remove planktonic cells and new biofilm growth medium was added. This washing step was repeated after 4 and 16 hours of incubation. After 24 h, the biofilm was staining using crystal violet and the absorbance was measured at 595 nm using a Tecan Infinite F200 Pro.

Rapid attachment of bacterial cells to a surface was analyzed as described previously [44]. Briefly, overnight cultures grown in BM2-medium were washed and diluted in BM2 medium containing 0.1% (w/v) casamino acids (CAA) to an OD_595nm_ of 1.0. One hundred μl of this suspension was used to inoculate each well of a microtiter plate. Cells were allowed to adhere for 60 min at 37°C prior to staining with crystal violet.

### RNA extraction, cDNA synthesis, and quantitative real-time PCR (qRT-PCR)

For analysis of virulence gene expression, overnight cultures of *P. aeruginosa* PA14 WT and the *typA* mutant were washed twice and resuspended in PBS buffer and adjusted to an OD_600_ of 2.0. For each bacterial cell suspension, 10 μl was mixed with washed amoeba cells of 2-day old *D. discoideum* cultures at a ratio of 3:1 bacteria to amoebae and the mixtures were plated on M9 agar plates. After incubation for 48 h at 22.5°C, cells were harvested from the agar plate surface, using an inoculation loop, and were resuspended in M9 medium supplemented with RNA protect reagent (Qiagen, Germany). To separate cells of *D. discoideum* from the bacterial cells, the mixtures were centrifuged for 1 min at 1,000 rpm and the supernatants containing the bacterial cells were used for RNA extraction.

RNA isolation, cDNA synthesis, and qRT-PCR analysis were performed as described previously [52] using the Power SYBR Green PCR Master Mix in an Abi 7300 Real Time PCR System (Applied Biosystems). All reactions were normalized to the house keeping gene *rpsL*. Experiments were repeated with three independent cultures.

## Competing interests

The authors declare that they have no competing interest.

## Authors’ contributions

OL and JO designed the experiments, supervised the research and wrote the paper. AN, ATYY, TR, BT, NS and MR did experiments and/or data analysis. All authors read and approved the final manuscript.
